# A rare coexistence of a non-dysraphic intradural lipoma and a cyst in the lumbar spine — A case report and literature review

**DOI:** 10.1016/j.ijscr.2024.110379

**Published:** 2024-10-01

**Authors:** Hala Khaddam, Abdulrahman Shbani, Obai Yousef, Ali Hussein, Iyas Salman, Issam Salman

**Affiliations:** a Faculty of Medicine, Tartous University, Tartous, Syria; b Department of Neurosurgery, Tartous University, Tartous, Syrian Arab Republic

**Keywords:** Intradural lipoma, Non-dysraphic lipoma, Intradural cysts, Arachnoid cyst, Extent of resection, Spinal cord

## Abstract

**Introduction:**

Spinal intradural lipomas are rare entities, particularly when not associated with spinal dysraphism. Their diagnosis is often delayed due to their slow growth and presentation in dorsal components. Although the association between lipomas and cysts has been previously noted, the coexistence of lipomas with fluid-filled cysts is exceedingly rare. We present a case that highlights the presentation, management, and outcomes of a patient with the unusual coexistence of these two lesions in the lumbar region.

**Case report:**

An eight-year-old female presented to our outpatient neurology clinic with complaints of weakness and severe pain in her lower limbs, accompanied by urinary retention and lumbar focal hypertrichosis. Magnetic resonance imaging (MRI) of the spine revealed a lipoma alongside a cyst at the level of the first two lumbar vertebrae. A subtotal resection of both lesions was performed; the cyst was drained, revealing a clear fluid, while the lipoma was partially resected due to significant adhesions to the spine. The patient achieved full recovery within a few days, as confirmed by neurological and radiological evaluations.

**Clinical discussion:**

The origin of non-dysraphic lipomas remains unclear, with several congenital theories proposed to explain their formation. While associations with various anomalies and cysts have been documented, the coexistence of intradural fluid-filled cysts is exceptionally uncommon. This case presents several potential explanations for this phenomenon. Typically, lipomas are asymptomatic until they cause signs of cord compression. However, the presence of associated entities can lead to earlier symptom onset. Surgical intervention often involves subtotal resection due to severe tumor adhesions, which can yield satisfactory outcomes despite the presence of residual tumor postoperatively.

**Conclusion:**

The occurrence of an intradural fluid-filled cyst in conjunction with a lipoma is rare and may represent an arachnoid cyst or a form of weakened spinal dysraphism linked to the controversial formation of lipomas. Illustrating this association is crucial, as the adhesions and indistinct boundaries between these lesions and the spinal cord complicate complete excision, necessitating subtotal resection, which appears to favor better outcomes.

## Highlights

• Non-dysraphic lipomas are significantly uncommon in comparison to their dysraphic counterparts, with most cases localized to the thoracic spine and only a few occurring in the lumbar spine.
• These slow-growing lesions present with periods of remission and recurrence. Symptoms include pain and gait difficulties. Lumbar lipomas affecting the cauda equina lead to flaccid paralysis and disturbances in sphincter control.
• The association between Lipomas and fluid-filled cysts is relatively rare.
• Magnetic Resonance Imaging (MRI) is the diagnostic modality of choice for both lipomas and arachnoid cysts. Fat suppression enhances the diagnostic accuracy to differentiate lipomas from other lesions.

## 1 Introduction

Intradural lipomas (ILs) of the spinal cord are uncommon, comprising approximately 1 % of all intraspinal tumors [[1], [2], [3]]. They are often associated with spinal dysraphism, as they are typically extradural and connected to a subcutaneous mass through a deformity in the posterior spinal components. This makes non-dysraphic lipomas even rarer [[1], [2], [3], [4]]. The latter are mostly localized in the thoracic spine and least commonly present in the lumbar spine [[1], [2], [3], [4]]. Lipomas usually manifest with gait difficulties, pain, and dysesthesia, reflecting slowly growing lesions characterized by periods of remission and recurrence [2,5]. The most common manifestations are ascending spasticity and numbness [2,5]. Flaccid paralysis and/or sphincter disturbances are peculiar to lower lipomas involving the lumbar spine and cauda equina [2]. They can be linked to abnormalities in fat metabolism or weight alterations, which can delay early diagnosis until signs of cord compression appear [4,6]. They have been reported to coexist with syringomyelia, hydromyelia, and several types of cysts [1,4,7]. However, the association with an intradural fluid-filled cyst is rare. This work has been conducted in accordance with the surgical case report (SCARE) guidelines [8].

## 2 Case presentation

An eight-year-old female with no significant medical history presented to the neurology clinic with congenital focal hypertrichosis in the lumbar region, urinary retention, and severe lower extremity pain that had progressively worsened over the past six months. She also reported diffuse lower limb numbness. The patient was referred to the clinic due to a sudden increase in pain severity. Neurological examination revealed gait disturbance and paraparesis (4/5 and 3/5 in the right and left lower limbs, respectively). No clonus or ataxia was observed.

**Fig. 1:**
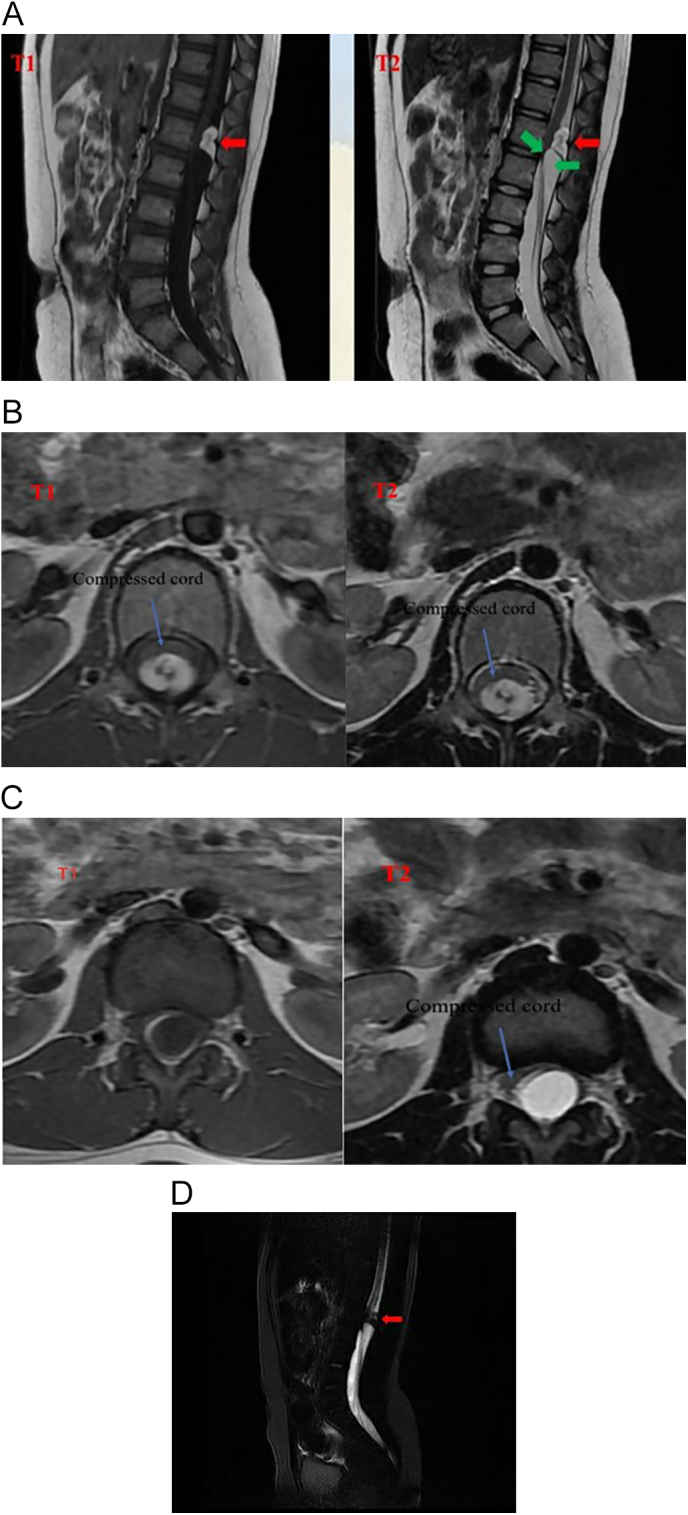
A, Preoperative sagittal T1-weighted MRI shows two lesions with different signals. The upper lesion was hyperintense, extending along the first lumbar vertebra on both T1 and T2 images corresponding to the characteristics of an intradural lipoma. The inferior lesion was hypointense on T1 image and hyperintense on T2 image indicating an intradural cyst. B, Axial MRI shows hyperintense lesion on both T1 and T2 images. C, Axial MRI shows a lesion that is hypointense on T1 and hyperintense on T2. D, T1-weighted MR image with fat suppression demonstrates the absence of the lesion.

Magnetic resonance imaging (MRI) of the spine was performed, revealing a dorsal mass at the first and second lumbar vertebrae (L1 and L2) consisting of two components. The first midline component was a 1.1 × 1.3 cm hyperintense lesion on both T1 and T2-weighted images at the L1 level, indicating an intradural lipoma (Fig. 1A-B). Fat suppression further supported this diagnosis (Fig. 1D). The second inferior component was a cyst that appeared hypointense on T1 and hyperintense on T2, clearly compressing the spinal cord (Fig. 1C).

**Fig. 2:**
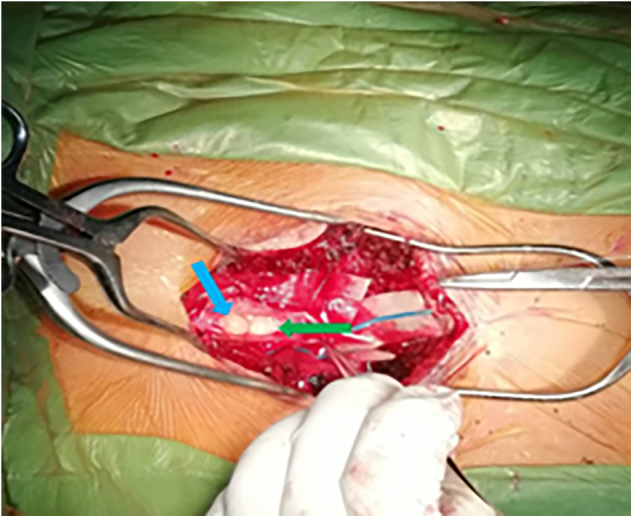
A picture during the surgery shows the Lipoma (blue arrow) and the cyst (green arrow).

The patient underwent subtotal resection of both lesions through a midline skin incision extending from T12 to L4, with laminectomy of L1 and L2. The dura was then incised at the midline, revealing the cyst and a yellowish tumor consistent with lipoma (Fig. 2). The cyst was drained, yielding clear fluid, and its wall was resected. A partial resection of the lipoma was performed with caution due to the absence of a neuromonitoring system in our facility and the difficulty in isolating the tumor from the spinal cord. Histopathological examination of the resected lipoma revealed mature adipose tissue with regular peripheral nuclei and associated fibrous tissue, with no signs of malignancy, thus confirming the diagnosis of fibrolipoma.

**Fig. 3:**
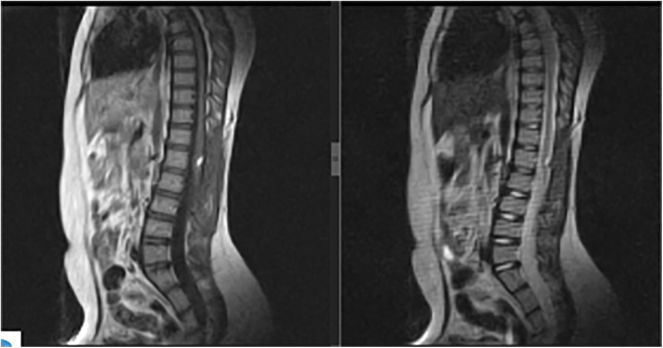
Postoperative MRI shows reduction of the lipoma with a slightly remained lesion.

The patient experienced full recovery within a few days post-surgery, free from complications, and her neurological examination returned to normal. A follow-up MRI scan showed only residual remnants of the lesions (Fig. 3). The patient has been followed for two years since the surgery and has reported no relevant complaints.

## 3 Discussion

The congenital origin of lipomas is often attributed to their association with various abnormalities and their presence at an early age [4]. The precise origin of intradural spinal lipomas remains unclear. While many researchers consider them to be of mesenchymal rather than neural origin [2], some cases have demonstrated discernible glial cells [9]. Several theories have been proposed to explain their genesis [1,10]. The “developmental error theory” is the most common and widely accepted, as it accounts for the dorsal positioning of the tumor and the absence of spinal dysraphism [1]. This theory may elucidate the aggregation of heterogeneous tissues within fat [1] and supports the classification of lipomas as hamartomas. However, instances of recurrence following subtotal resection suggest a neoplastic nature for these lesions [9]. Various types of cysts have also been reported as coexisting lesions [1,5,7,11].

The final diagnosis of our cyst carries several possibilities. Naidich et al. proposed a hypothesis explaining dysraphism formation [12]. This suggestion aligns with the development of non-dysraphic lipomas but involves a less severe mechanism, excluding abnormalities in vertebrae and soft tissues [6]. Given that dysraphic lipomas are more frequently encountered in the lumbosacral region and are often associated with cutaneous markers [13], the cyst in our case may represent a form of mild spinal dysraphism. This hypothesis is supported by another similar case that did not specify the cyst's type [7].

On the other hand, arachnoid cysts can be classified as intradural or extradural, further divided into primary and secondary lesions [14,15]. Thus, this cyst might be secondary to fibrous adhesions and abnormal cerebrospinal fluid (CSF) flow, as evidenced by the absence of septations in the lumbar region [16]. Alternatively, the cyst could be primary, given that the coexistence of intradural lipoma and arachnoid cyst has been documented alongside other congenital malformations such as melanocytosis, spina bifida, and cloacal [[17], [18], [19]]. Moreover, arachnoid cysts have been linked to neural tube defects [19], suggesting a probable congenital association between these two lesions. Overall, the radiological features—characterized by a T1-hypointense and T2-hyperintense lesion containing clear fluid—combined with the lack of biopsy confirmation, point towards a probable diagnosis of an arachnoid cyst.

Lipomas are typically asymptomatic for extended periods and are often diagnosed in the second or third decade of life [20]. However, individuals with cutaneous stigmata tend to receive diagnoses at earlier ages, averaging around 9 months [21]. When symptoms do arise, lipomas present a clinical picture consistent with cord compression, including neurological deficits and pain. Notably, pain associated with lipomas is usually localized, whereas pain from arachnoid cysts tends to be radicular [2,5,20,22], raising suspicion that the cyst may be arachnoid. The mean duration of symptoms related to lipomas typically exceeds three years, although a small number of cases present symptoms within less than a year [2,23]. Furthermore, while our patient exhibited focal hypertrichosis, this sign was underestimated until neurological deterioration occurred.

MRI is the diagnostic modality of choice for both lipomas and arachnoid cysts [1,24]. The lesion's location and associated entities can limit diagnostic possibilities; however, fat suppression enhances accuracy [1,25]. Lipomas are occasionally associated with other intradural cysts such as dermoid, epidermoid, and neurenteric cysts [3,16]. Diffusion-weighted imaging (DWI) can aid in differentiating them from epidermoid cysts [26]. Kinematic MRI and myelography can reveal communication between the cyst and the subarachnoid space [24,26]. Some authors have confirmed diagnoses through biopsy [15,27,28], while others have relied on MRI and intraoperative findings [14,20,29]. Nevertheless, when feasible, biopsy is recommended to exclude tumoral types [30]. In our case, the lipoma was confirmed through biopsy, while we relied on MRI and surgical findings to identify the cyst.

Total excision has been reported to yield the best outcomes [4]. However, Ammerman et al. found that total removal is associated with higher morbidity rates compared to subtotal resection [5]. Despite these guidelines, lipomas often adhere to surrounding tissues via ventral fibrous strands, complicating the delineation of a clear cleavage plane between the tumor and the spine [4,10]. This challenge frequently necessitates subtotal resection. Additionally, a case series involving five non-dysraphic patients demonstrated that the extent of excision does not significantly impact postoperative outcomes [12]. The results of lipoma resection depend on various factors, including the amount excised, the severity of symptoms, and the duration of preoperative symptoms [3,12]. Overall, outcomes can range from unchanged status to partial improvement in specific symptoms [1,14,22], with complete recovery being rare. Conversely, the prognosis for arachnoid cysts is generally favorable [14,15,20].

Recurrence is a significant concern, with the interval between surgery and recurrent lipomas varying from one to ten years; some lipomas may recur multiple times [1,12]. Higher recurrence rates are associated with subtotal resections [31]. In our case, although a slight rim of both lesions was left, the patient achieved full recovery, confirmed through subsequent radiological and clinical evaluations. This supports the notion that the extent of resection may not correlate with postoperative outcomes. Nevertheless, due to the potential for recurrence over varying intervals, ongoing evaluation is essential.

## 4 Conclusion

We present a case of a non-dysraphic intradural fibrolipoma partially involving the cord, accompanied by an intradural cyst in the lumbar region. The combination of intradural lipomas and fluid-filled cysts is rare and has controversial explanations, including weakened spinal dysraphism or associated arachnoid cysts. Understanding the embryological mechanisms underlying lipoma formation, potential associations with other anomalies, and how lipomas can lead to abnormal cerebrospinal fluid (CSF) flow resulting in secondary cysts is crucial. Furthermore, we conclude that subtotal resection is preferable in cases with dense adhesions and partial intramedullary lipomas, demonstrating favorable outcomes despite the amount removed and highlighting the necessity for periodic clinical and radiological evaluations.

## 5 Methods

The work has been reported in line with SCARE criteria.

## Statement on any prior presentation

Not available.

## Ethical approval

Our institution (Tartous University) does not require ethical approval for reporting individual cases or case series.

## Funding

The authors received no financial support for the research, authorship, and/or publication of this article.

## Author contribution

HK, AS, OY, AH, IS drafted the manuscript. IS^6^ supervised the patient's examination, treatment, and follow-up. All authors have read and approved the final manuscript.

## Guarantor

**Hala Khaddam** accepts full responsibility for the work, had access to the data, and controlled the decision to publish.

## Research registration number

Not applicable since it is a case report.

## Declaration of competing interest

The authors declare that they have no known competing financial interest or personal relationship that could have appeared to influence the work reported in this paper.

## References

[1] Kabir S.M.R., Thompson D., Rezajooi K., Casey A.T.H. (2010). Non-dysraphic intradural spinal cord lipoma: case series, literature review and guidelines for management. Acta Neurochir..

[2] Giuffr R. (1966). Intradural spinal lipomas: review of the literature (99 cases) and report of an additional case. Acta Neurochir..

[3] Chen K.-Y., Osorio J., Rivera J., Chou D. (2019). Intramedullary and extramedullary thoracic spinal lipomas without spinal dysraphism: clinical presentation and surgical management. World Neurosurg..

[4] Caram P.C., Scarcella G., Carton C.A. (1957). Intradural lipomas of the spinal cord: with particular emphasis on the “intramedullary” lipomas. J. Neurosurg..

[5] Ammerman B.J., Henry J.M., De Girolami U., Earle K.M. (1976). Intradural lipomas of the spinal cord: a clinicopathological correlation. J. Neurosurg..

[6] Bhatoe H.S., Singh P., Chaturvedi A., Sahai K., Dutta V., Sahoo P.K. (2005). Nondysraphic intramedullary spinal cord lipomas: a review. FOC.

[7] Falavigna A., Segatto A.C., Salgado K. (2001). A rare case of intramedullary lipoma associated with cyst. Arq. Neuropsiquiatr..

[8] C. Sohrabi, G. Mathew, N. Maria, A. Kerwan, T. Franchi, R.A. Agha, The SCARE 2023 guideline: updating consensus Surgical CAse REport (SCARE) guidelines, Int. J. Surg. 109 (5) (May 1 2023) 1136–1140. doi:10.1097/JS9.0000000000000373, (n.d.). PMC10389401 37013953

[9] Muthukumar N. (2009). Congenital spinal lipomatous malformations: part II—clinical presentation, operative findings, and outcome. Acta Neurochir..

[10] Kim C.H., Wang K.-C., Kim S.-K., Chung Y.-N., Choi Y.L., Chi J.G., Cho B.-K. (2003). Spinal intramedullary lipoma: report of three cases. Spinal Cord.

[11] Rauzzino M.J., Tubbs R.S., Alexander E., Grabb P.A., Oakes W.J. (2001). Spinal neurenteric cysts and their relation to more common aspects of occult spinal dysraphism. FOC.

[12] Fleming K.L., Davidson L., Gonzalez-Gomez I., McComb J.G. (2010). Nondysraphic pediatric intramedullary spinal cord lipomas: report of 5 cases. PED.

[13] Finn M.A., Walker M.L. (2007). Spinal lipomas: clinical spectrum, embryology, and treatment. FOC.

[14] Lee H.-J., Cho D.-Y. (2001). Symptomatic spinal Intradural arachnoid cysts in the pediatric age group: description of three new cases and review of the literature. Pediatr. Neurosurg..

[15] Guzel A., Tatlı M., Yılmaz F., Bavbek M. (2007). Unusual presentation of cervical spinal intramedullary arachnoid cyst in childhood: case report and review of the literature. Pediatr. Neurosurg..

[16] Klekamp J. (2017). A new classification for pathologies of spinal meninges—part 2: primary and secondary intradural arachnoid cysts. NEUROSURGERY.

[17] Kasantikul V., Shuangshoti S., Pattanaruenglai A., Kaoroptham S. (1989). Intraspinal melanotic arachnoid cyst and lipoma in neurocutaneous melanosis. Surg. Neurol..

[18] Fujimura M., Kusaka Y., Shirane R. (2003). Spinal lipoma associated with terminal syringohydromyelia and a spinal arachnoid cyst in a patient with cloacal exstrophy. Childs Nerv. Syst..

[19] Rabb C.H., McComb J.G., Raffel C., Kennedy J.G. (1992). Spinal arachnoid cysts in the pediatric age group: an association with neural tube defects. J. Neurosurg..

[20] Kumar K., Malik S., Schulte P.A. (2003). Symptomatic spinal arachnoid cysts: report of two cases with review of the literature. Spine.

[21] Massimi L., Feitosa Chaves T.M., Legninda Sop F.Y., Frassanito P., Tamburrini G., Caldarelli M. (2019). Acute presentations of intradural lipomas: case reports and a review of the literature. BMC Neurol..

[22] Fujiwara F., Tamaki N., Nagashima T., Nakamura M. (1995). Intradural spinal lipomas not associated with spinal dysraphism. Neurosurgery.

[23] Ahmed O., Zhang S., Thakur J., Nanda A. (2015). Nondysraphic intramedullary cervical cord lipoma with exophytic component: case report. J Neurol Surg Rep.

[24] Silbergleit R., Brunberg J.A., Patel S.C., Mehta B.A., Aravapalli S.R. (1998). Imaging of spinal intradural arachnoid cysts: MRI, myelography and CT. Neuroradiology.

[25] Beall D.P., Googe D.J., Emery R.L., Thompson D.B., Campbell S.E., Ly J.Q., DeLone D., Smirniotopoulos J., Lisanti C., Currie T.J. (2007). Extramedullary Intradural spinal tumors: a pictorial review. Curr. Probl. Diagn. Radiol..

[26] Yuen J., McGavin L., Adams W., Haden N. (2021). Intradural symptomatic arachnoid cyst formation following non-instrumented lumbar decompression. Br. J. Neurosurg..

[27] Nath P.C., Mishra S.S., Deo R.C., Satapathy M.C. (2016). Intradural spinal arachnoid cyst: a long-term postlaminectomy complication: a case report and review of the literature. World Neurosurg..

[28] Medved F., Seiz M., Baur M.-O., Neumaier-Probst E., Tuettenberg J. (2009). Thoracic intramedullary arachnoid cyst in an infant: case report. PED.

[29] Ichinose T., Miyashita K., Tanaka S., Oikawa N., Oishi M., Nambu I., Kinoshita M., Nakada M. (2020). Recurrent spinal intramedullary arachnoid cyst: case report and literature review. World Neurosurg..

[30] Agnoli A.L., Schönmayr R., Laun A. (1982). Intraspinal arachnoid cysts. Acta Neurochir..

[31] Pang D. (2019). Surgical management of complex spinal cord lipomas: how, why, and when to operate. A review: JNSPG 75th anniversary invited review article. J. Neurosurg. Pediatr..

